# Prognostic value of decreased long non-coding RNA TUSC7 expression in some solid tumors: a systematic review and meta-analysis

**DOI:** 10.18632/oncotarget.18496

**Published:** 2017-06-15

**Authors:** Na Li, Meilan Yang, Ke Shi, Wei Li

**Affiliations:** ^1^ Department of Pathology, The First Affiliated Hospital of Hunan University of Medicine, Huaihua, Hunan Province, China; ^2^ Department of Geriatrics, Xiangya Hospital of Central South University, Changsha, Hunan Province, China

**Keywords:** TUSC7, cancer, clinical outcome, prognosis

## Abstract

Accumulating evidences indicated that tumor suppressor candidate 7 (TUSC7) is a putatively tumor suppressor gene in various tumors. We carried out current systematic review and meta-analysis to explore the decreased expression of TUSC7 associate with prognostic and clinicopathological characteristic in cancer patients. A literature collection search in the online electronic databases PubMed, Embase, Web of Science, and CNKI was conducted to obtain eligible studies (up to February 20, 2017). A total of nine studies comprise 757 patients were identified and included in present meta-analysis based on the selection and inclusion criteria. Overall, low expression of TUSC7 was associated with significantly unfavorable overall survival (OS) (HR = 2.90, 95% CI: 2.12–3.98, *P* < 0.001), disease free survival (DFS) (HR = 2.00, 95% CI: 1.49–2.68, *P* < 0.001) and disease-specific survival (DSS) (HR = 2.57, 95% CI: 1.23–5.39, *P* = 0.012) in tumors patients. Moreover, we also found that down-regulation of TUSC7 associated with distant metastasis (OR = 2.85, 95% CI: 1.46–5.55, *P* = 0.002) and larger tumor size (OR = 0.41, 95% CI: 0.23–0.72, *P* = 0.002). Our meta-analysis demonstrated that cancers patients detected with low TUSC7 expression were more prone to develop distant metastasis. TUSC7 might act as a potentially and promising common prognostic markers in some solid tumors.

## INTRODUCTION

Numerous studies have indicated that long noncoding RNAs (lncRNAs) are dysregulation in a variety of diseases, including cancer [1–5]. LncRNAs participate in regulating a wide range of biological processes at transcriptional and posttranscriptional levels [6], which play a key role in tumor genesis, progression and prognosis of cancers [2, 7–9]. Tumor suppressor candidate 7 (TUSC7), also called LSAMP antisense RNA3 (LSAMP-AS3) or lncRNA LOC285194, is a newly determined lncRNA, comprising 4 exons with length of about 2.1 kb and is situated at chromosome region of 3q13.31 [10]. It was first reported as a decreased expression gene in human osteosarcoma; depleting this lncRNA contributed to normal osteoblasts proliferation by regulating cell apoptotic and cell cycle transcripts as well as VEGF receptor 1 [10]. In addition, lncRNA TUSC7 serve as a p53-regulated tumor suppressor in gastric and colon cancer cells, in part by inhibiting miR-23b and miR-211 to inhibit cell growth, respectively [11–13]. Thence, TUSC7 is considered to be a tumor suppressor gene.

For the last 5 years, many reports have revealed that decreased TUSC7 expression is correlated with unfavorable overall survival (OS) in various tumors, including non-small-cell lung cancer (NSCLC) [7], colorectal cancer (CRC) [13, 14], esophageal squamous cell carcinoma (ESCC) [15], osteosarcoma (OSA) [16] and so on. Besides, low expression of TUSC7 are poor prognostic factors for disease free survival (DFS) in human gastric cancer (GC) [12], hepatocellular carcinoma (HCC) [17], CRC [14], ESCC [15], as well as NSCLC [7]. Studies also continue to indicate that TUSC7 loss plays a role in the poor disease-specific survival (DSS) in patients with gastric cancer [12] and colorectal cancer [14]. Furthermore, decreased TUSC7 expression is also related to tumor clinical characteristics [18, 19]. In addition, serum TUSC7 level displayed a high diagnostic value for patients with colorectal cancer [20]. These results are consistent with lncRNA TUSC7 act as a potential common tumor suppressor and prognosis marker.

However, because of the limitations associated with the research design and sample size, single study may be inaccurate and inadequate. As such, it is necessary to systematically explore the potential clinical values of TUSC7 in cancers. Thus far, no meta-analysis has yet been conducted to assess the relation between TUSC7 expression and clinical outcomes in various tumors. Therefore, we collected all relevant publications and performed present systematic review and meta-analysis to detect the clinical values of TUSC7 expression level in various tumors. We mainly discussed the expression of TUSC7 associate with prognosis, metastasis and clinicopathological characteristics of cancer patients. It aimed to more precisely evaluate the association between TUSC7 expression and clinical consequence of human cancer.

## RESULTS

### Study characteristics

**Figure 1:**
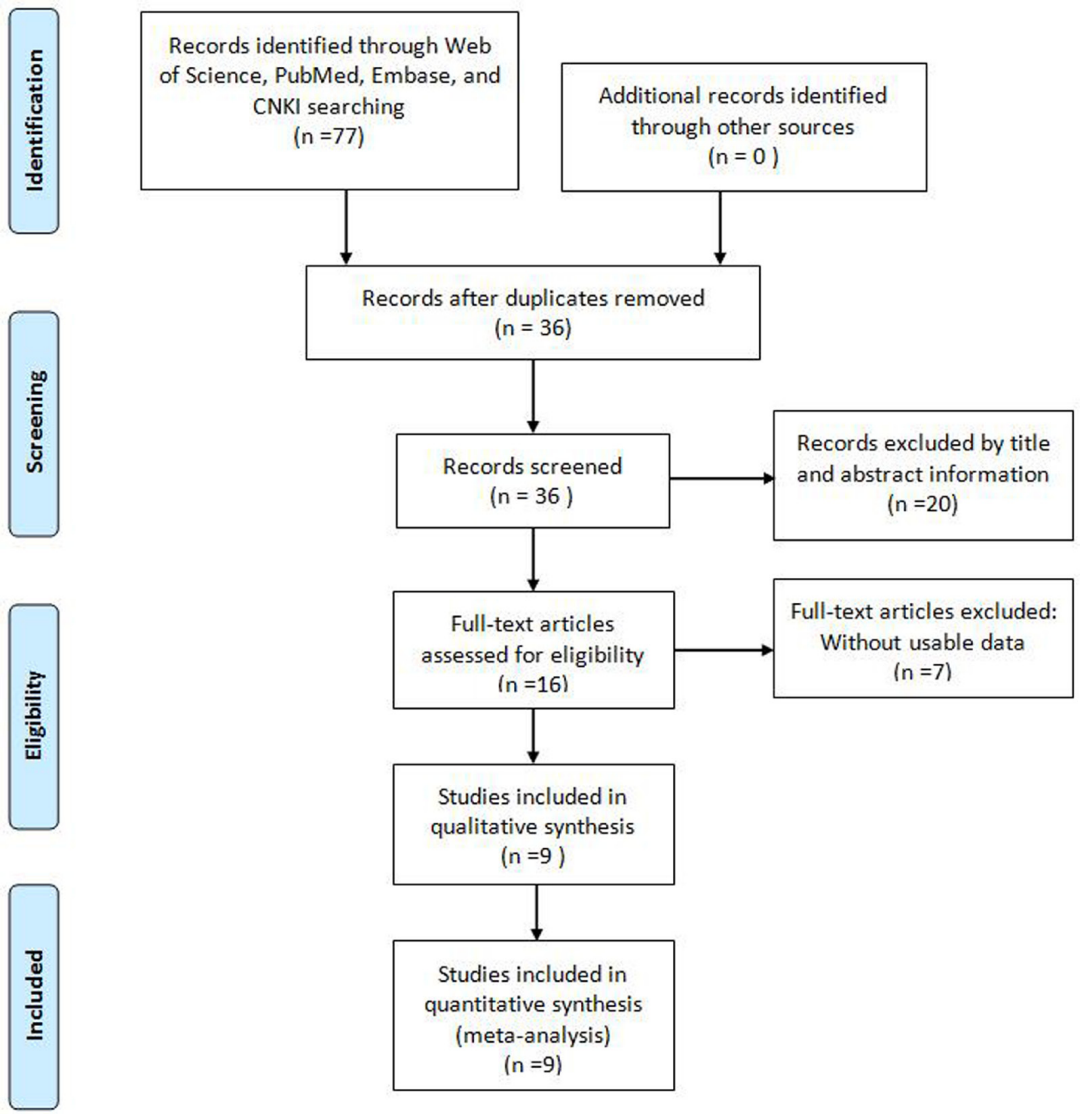
Flow diagram of the literature search and selection

As shown in the flowchart (Figure 1), a total of 77 reports were searched from Embase, Web of Science, PubMed, and CNKI. After the duplicates were excluded, 36 records were preserved. After reviewing the title and abstracts, 20 records were removed. After further inspection of the full text carefully, a total of 9 studies were ultimately identified according to the criteria [7, 12–19]. The 9 eligible studies comprise 757 patients and all came from China. Among these cancers included in this meta-analysis derived from eight different tumor types: pancreatic ductal adenocarcinoma [19], gastric cancer (GC) [12], esophageal squamous cell carcinoma (ESCC)[15], osteosarcoma (OSA) [16], hepatocellular carcinoma (HCC) [17], non-small-cell lung cancer (NSCLC) [7], colorectal cancer (CRC) [13, 14], and Glioma [18]. All of the TUSC7 expression level were detected by qRT-PCR and divided into high expression and low expression groups. All tumor patients were diagnosed based on pathology. The main characteristics of the include studies were summarized in Table 1.

**Table 1:** Main characteristics of the include studies for prognostic

Study	Region	Tumor type	Sample size	Test method	Cut-off	Outcome measure	HR estimation	Follow-up (months)
Qi 2013	China	CRC	81	qRT-PCR	mean value	DFS/DSS	Directly	~60
Ding 2014	China	PDAC	85	qRT-PCR	mean value	OS	Directly	~60
Tong 2014	China	ESCC	142	qRT-PCR	median value	OS/DFS	Directly	~36
Qi 2015	China	GC	78	qRT-PCR	mean value	DFS/DSS	Directly	Over 60
Cong 2016	China	OSA	82	qRT-PCR	NA	OS	Directly	Over 110
Shang 2016	China	Glioma	39	qRT-PCR	NA	OS	Directly	~40
Wang Y 2016	China	HCC	75	qRT-PCR	mean value	OS/DFS	Directly	~36
Wang Z 2016	China	NSCLC	112	qRT-PCR	median value	OS/DFS	Directly	Over 60
Xu 2017	China	CRC	63	qRT-PCR	mean value	OS	Directly	Over 100

PDAC: pancreatic ductal adenocarcinoma; GC: gastric cancer; ESCC: esophageal squamous cell carcinoma; OSA: osteosarcoma; HCC: hepatocellular carcinoma; NSCLC: non-small-cell lung cancer; CRC: colorectal cancer; OS: overall survival; DFS: disease free survival; DSS: disease-specific survival; qRT-PCR: quantitative real-time PCR; NA: not available.

### Meta-analysis results

### Association between TUSC7 and OS

**Figure 2:**
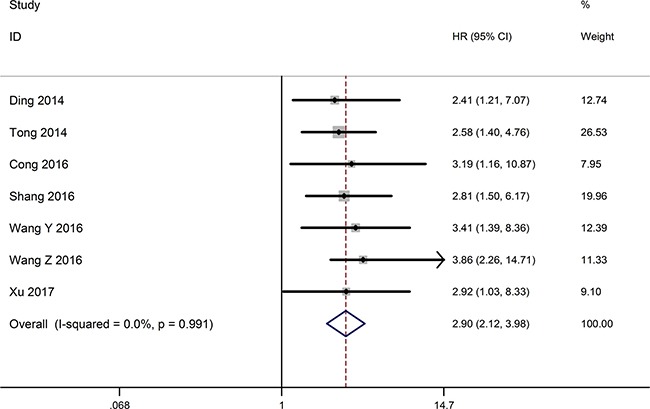
Forest plot for the relationships between decreased TUSC7 expression and OS

The relationship between the expression level of TUSC7 and overall survival (OS) was detected in seven researches including 598 patients (Table 1). The fixed effects model was applied to calculate the overall hazard ratios (HRs) because of no heterogeneity among studies (I^2^ = 0.0%, *P* = 0.991). The pooled result (HR = 2.90, 95% CI: 2.12–3.98, *P* < 0.001) indicated that decreased TUSC7 expression exhibited a significantly shorter OS than those with high TUSC7 expression (Figure 2). Consequently, decreased TUSC7 expression associated with a worse survival in tumor patients.

### Association between TUSC7 and DFS/DSS

**Figure 3:**
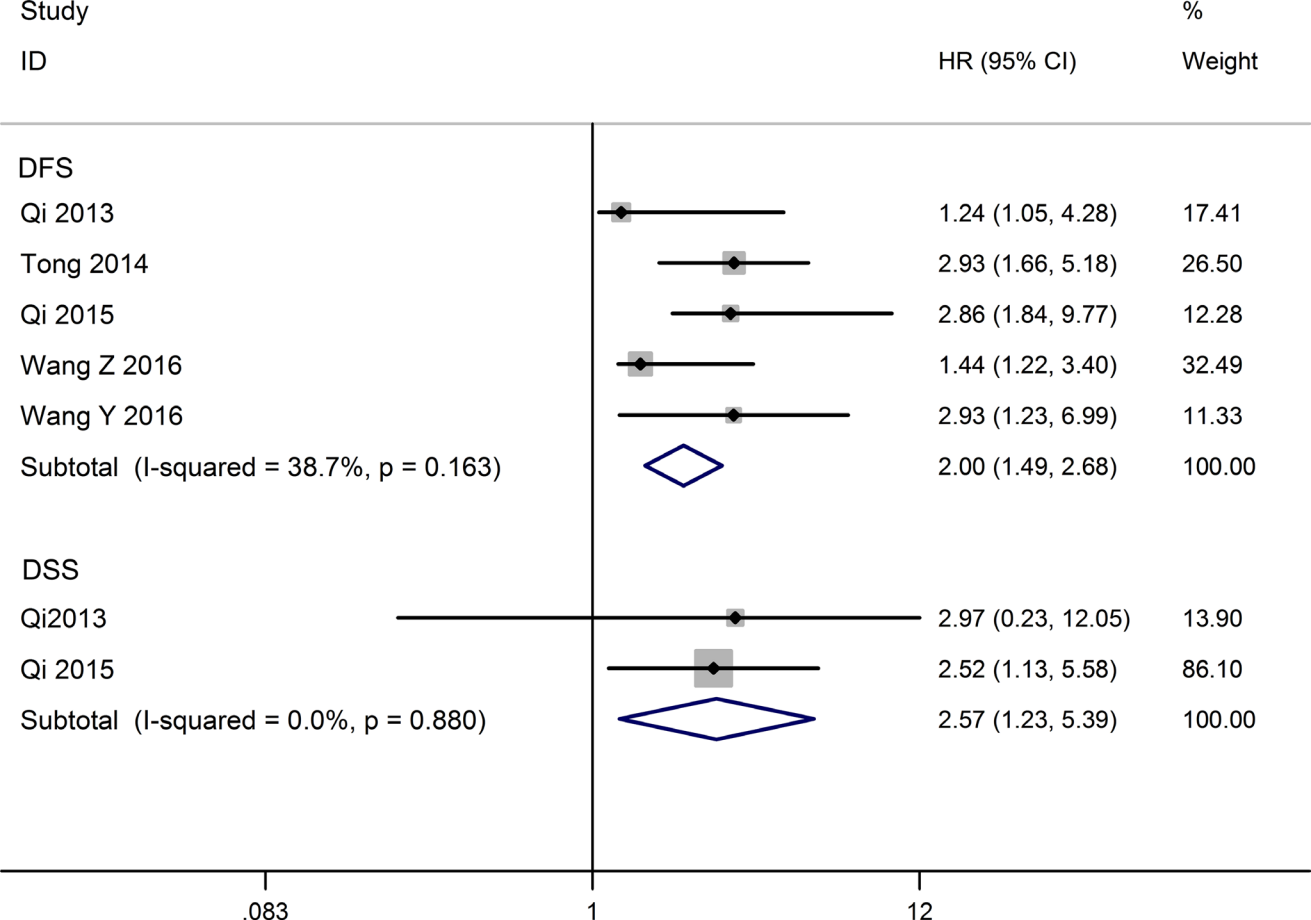
Forest plot for the relationships between decreased TUSC7 expression and DFS/DSS

Five studies include a total of 488 patients reported HRs for disease free survival (DFS). No significant heterogeneity across these studies (I^2^ = 38.7%, *P* = 0.163), so the fixed-effects model was used to pool the HRs. The aggregated results revealed that the low TUSC7 expression was significantly correlation with poor DFS (HR = 2.00, 95% CI: 1.49–2.68, *P* < 0.001) (Figure 3). Only two eligible articles reporting a total of 159 patients were offered data to analyze disease-specific survival (DSS). Because of the small heterogeneity (I^2^ = 0%, *P* = 0.880), the fixed effect model was adopted. The summarized result indicated decreased TUSC7 expression represents a poor clinical outcome for DSS (HR =2.57, 95% CI: 1.23–5.39, *P* = 0.012) (Figure 3).

### Associations between TUSC7 and clinicopathological parameters

From the pooled results showed in Table 2, it found that decreased TUSC7 expression was positively correlated with distant metastasis (OR = 2.85, 95% CI: 1.46–5.55, *P* = 0.002, fixed effects model) and tumor size (OR = 0.41, 95% CI: 0.23–0.72, *P* = 0.002, fixed effects model). However, no significant correlations were observed between the decreased TUSC7 expression and other clinicopathologic parameters involving lymph node metastasis, gender, clinical stage and invade depth (*P* > 0.05). Due to insufficient data, we were unable to detect the association between low TUSC7 expression and other clinicopathological factors.

**Table 2:** Odds ratio for the association between decreased TUSC7 expression and clinicopathological parameters

Clinicopathological parameter	Patients size	OR (95% CI)	*P* value	Heterogeneity		
				*I^2^*	*P_h_*	Model
Gender (Male vs. Female)	495	0.74 (0.51–1.06)	0.102	0.0%	0.456	Fixed effects
Clinical stage ( I/II vs. III/IV)	495	0.84 (0.23–3.14)	0.800	91.1.0%	< 0.001	Random effects
Lymph node metastasis (Yes vs. No)	420	2.31 (0.92–5.82)	0.076	80.2%	0.002	Random effects
Distant metastasis (Yes vs. No)	223	2.85 (1.46–5.55)	**0.002**	0.0%	0.644	Fixed effects
Depth of invasion (T1/T2 vs. T3/T4)	308	0.80 (0.49–1.29)	0.358	0.0%	0.725	Fixed effects
Tumor size (< 5 cm vs. ≥ 5 cm)	217	0.41 (0.23–0.72)	**0.002**	39.5%	0.199	Fixed effects

### Sensitivity analysis

**Figure 4:**
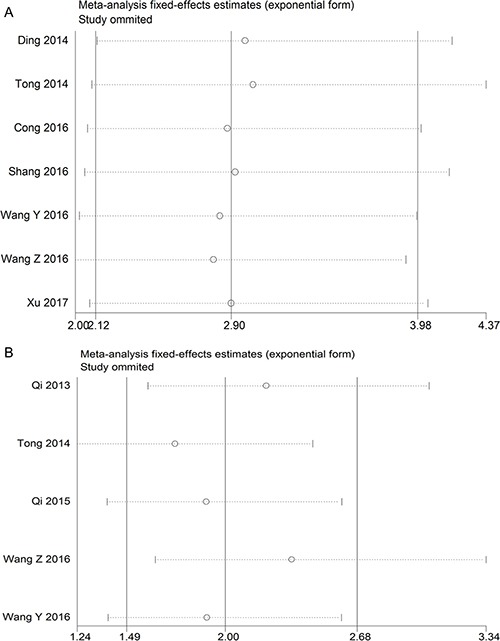
The sensitivity analysis for the meta-analysis of OS or DFS in tumor patients (**A**) OS group; (**B**) DFS group.

To evaluate the robustness of the summarized results in this study, a sensitivity analysis was conducted by successively excluding each individual study from the pooled analysis. The result of the relationship between TUSC7 expression and OS or DFS was not significantly influenced after excluding any study, suggesting that the results were robust (Figure 4). Considering that the small number of studies, sensitivity analysis of other groups has not been carried out.

### Evaluation of publication bias

**Figure 5:**
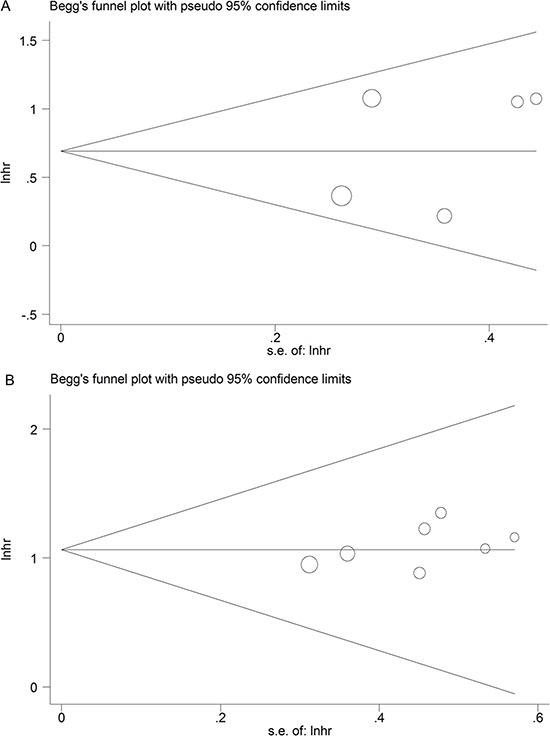
Funnel plot analysis of potential publication bias for meta-analysis (**A**) DFS group; (**B**) OS group.

The publication bias of this meta-analysis was evaluated by Begg's funnel plot analysis. There was no obvious publication bias in both DFS group (*Pr* >|z| = 0.462) and OS group (*Pr* > |z| = 0.133) by Begg's test (Figure 5).

## DISCUSSION

A lot of studies have proved that dysregulated expression of lncRNAs is closely related to tumorigenesis and cancer progression [21–24]. TUSC7 was a newly identified ncRNA gene, reported to be decreased expression in various cancer cells and tumor tissues as a tumor suppressor [7, 13, 16–19]. TUSC7 influences the proliferation, apoptosis, and cell cycle transcripts in normal osteoblasts [10]. Moreover, by repressing miR-23b transcription, TUSC7 inhibited cellular proliferation, migration and invasion and promoted cellular apoptosis in gastric cancer and glioma cells [12, 18]. Additionally, TUSC7 acts as a molecular sponge of miR-10a and suppresses EMT through decrease the expression of Eph tyrosine kinase receptor A4 in human hepatocellular carcinoma [17]. In addition, previous studies have reported that down-regulation of TUSC7 was associated with chemoresistance in ESCC [15]. Therefore, further explore the relationship between TUSC7 and cancer is absolutely necessary.

Here we implemented present meta-analysis to evaluate the relationship of TUSC7 expression levels with prognosis results and clinicopathological parameter in cancer patients. To the best of our knowledge, this is the first meta-analysis offering comprehensive insights into the prognostic value of TUSC7 in human cancer. There were altogether 9 eligible studies met the selection requirements in our meta-analysis. The survival data comprised OS, DFS, DSS. The results indicated that decreased TUSC7 expression was significantly related to unfavorable clinical prognosis in patients with various tumor types. Firstly, the pooled results demonstrated that the patients with low TUSC7 expression exhibited a shorter OS than those with high TUSC7 expression. Secondly, the patients with decreased TUSC7 significantly related to unfavorable DFS or DSS. These results showed that TUSC7 could serve as a potential independent predictive biomarker for OS or DFS or DSS in tumor patients. Thirdly, the aggregated results showed that decreased TUSC7 expression was positively correlated with distant metastasis and larger tumor size. In other words, cancer patients with low TUSC7 expression in tumor tissues were more prone to develop DM. Finally, no significant correlations were observed between the decreased TUSC7 expression and lymph node metastasis or gender or clinical stage or invade depth. However, some studies reported that decreased expression of TUSC7 was closely associated with more lymphnode metastasis [14, 15, 19]. To explore whether the heterogeneity affect the pooled results, we found decreased TUSC7 expression was positively associated with LNM (OR = 3.29, 95% CI: 2.05–5.28, *P* < 0.001, fixed effects model) after exclude a datasets of LNM from Wang et al. [7]. Because of the small size of the study, this conclusion may be insufficiently convincing. Therefore, further researches are needed to confirm this completion.

Although TUSC7 was found to be significantly correlated with the prognosis of cancer patients, some limitations should be concerned for explaining the results of this meta-analysis. For instance, only nine studies included in our meta-analysis and patients were all Asians from China, thence, our results may be suitable only to this Chinese population. Additionally, the cut-off values of decreased TUSC7 expression not consistent. Finally, there was significant heterogeneity in some clinicopathological parameters analysis. Thus, the results of this meta-analysis should be confirmed by future studies with well-designed and larger-size.

In conclusion, our meta-analysis offers evidence that low TUSC7 expression is a risk factor for distant metastasis in diverse cancers. TUSC7 could act as a potentially and promising prognostic markers in human some solid tumors.

## MATERIALS AND METHODS

### Publication search

We searched online electronic databases Embase, PubMed, Web of Science and CNKI (up to February 20, 2017) to obtain the publications about lncRNA TUSC7 expression. The search terms were as follows: “TUSC7” [Title/Abstract] OR “tumor suppressor candidate 7” [Title/Abstract] OR “LOC285194” [Title/Abstract] OR “LSAMP-AS3” [Title/Abstract] OR “LSAMP antisense RNA3” [Title/Abstract]. Only include English or Chinese publications in this study.

### Study inclusion and exclusion criteria

The inclusion criteria of present meta-analysis as follows: (1) article researched the association with TUSC7 expression and cancer prognosis; (2) cancer patients were divided into two groups: high TUSC7 expression or low TUSC7 expression; (3) reporting related clinical parameters, such as lymph node metastasis and distant metastasis; (4) providing sufficient data for the computation of OR, HR and corresponding 95% CI. Exclusion criteria of this meta-analysis are following: (1) nonhuman research; (2) duplicate publications; (3) case reports, letters, expert opinions, editorials and reviews; (4) studies without available data.

### Quality assessment and data extraction

Two investigators (Na Li and Wei Li) using the Newcastle-Ottawa Quality Assessment Scale (NOS) to assess the quality of all eligible studies independently, which were reported in previously studies [25, 26]. All included studies were considered to be of high quality based on NOS. The data from each eligible study were extracted and reviewed by two authors (Wei Li and Ke Shi) independently. Disagreements were discussed with third researcher (Na Li) to reach a consensus. The following information from every study was collected: first author's name, publication date, study region, tumor type, detection method of TUSC7 expression, criteria of TUSC7 high expression, total patients amount, clinicopathological parameter, and survival analysis, follow-up period, HR and corresponding 95% CI. If the available survival data not shown in articles, corresponding authors were contacted to collect these data or extracted from graphical survival plots and HRs were estimated.

### Statistical methods

All statistical analysis of the present study was conducted by STATA SE 12.0 software (Stata, College Station, Texas). The heterogeneity of pooled HRs or ORs among included studies was detected using the Q-statistic test and chi-squared based. The fixed-effects model was used to pool the results when the included studies with small heterogeneity (I^2^ < 50%, *P* > 0.1). Otherwise, a random effects model was applied (I^2^ > 50%, *P* < 0.1). A sensitivity analysis was executed to evaluate the robustness of the overall results. The “Begg's funnel plot” was used to assess potential publication bias. All the *P*-values less than 0.05 were regarded as statistical significance.

## References

[1] Qian Y, Liu D, Cao S, Tao Y, Wei D, Li W, Li G, Pan X, Lei D (2017). Upregulation of the long noncoding RNA UCA1 affects the proliferation, invasion, and survival of hypopharyngeal carcinoma. Mol Cancer.

[2] Adams BD, Parsons C, Walker L, Zhang WC, Slack FJ (2017). Targeting noncoding RNAs in disease. J Clin Invest.

[3] Beermann J, Piccoli MT, Viereck J, Thum T (2016). Non-coding RNAs in Development and Disease: Background, Mechanisms, and Therapeutic Approaches. Physiol Rev.

[4] Parikshak NN, Swarup V, Belgard TG, Irimia M, Ramaswami G, Gandal MJ, Hartl C, Leppa V, Ubieta LT, Huang J, Lowe JK, Blencowe BJ, Horvath S (2016). Genome-wide changes in lncRNA, splicing, and regional gene expression patterns in autism. Nature.

[5] Chang S, Chen B, Wang X, Wu K, Sun Y (2017). Long non-coding RNA XIST regulates PTEN expression by sponging miR-181a and promotes hepatocellular carcinoma progression. BMC Cancer.

[6] Chen LL (2016). Linking Long Noncoding RNA Localization and Function. Trends Biochem Sci.

[7] Wang Z, Jin Y, Ren H, Ma X, Wang B, Wang Y (2016). Downregulation of the long non-coding RNA TUSC7 promotes NSCLC cell proliferation and correlates with poor prognosis. Am J Transl Res.

[8] Chandra Gupta S, Nandan Tripathi Y (2017). Potential of long non-coding RNAs in cancer patients: From biomarkers to therapeutic targets. Int J Cancer.

[9] Li X, Cao Y, Gong X, Li H (2017). Long noncoding RNAs in head and neck cancer. Oncotarget.

[10] Pasic I, Shlien A, Durbin AD, Stavropoulos DJ, Baskin B, Ray PN, Novokmet A, Malkin D (2010). Recurrent focal copy-number changes and loss of heterozygosity implicate two noncoding RNAs and one tumor suppressor gene at chromosome 3q13.31 in osteosarcoma. Cancer Res.

[11] Liu Q, Huang J, Zhou N, Zhang Z, Zhang A, Lu Z, Wu F, Mo YY (2013). LncRNA loc285194 is a p53-regulated tumor suppressor. Nucleic Acids Res.

[12] Qi P, Xu MD, Shen XH, Ni SJ, Huang D, Tan C, Weng WW, Sheng WQ, Zhou XY, Du X (2015). Reciprocal repression between TUSC7 and miR-23b in gastric cancer. Int J Cancer.

[13] Xu J, Zhang R, Zhao J (2017). The Novel Long Noncoding RNA TUSC7 Inhibits Proliferation by Sponging MiR-211 in Colorectal Cancer. Cell Physiol Biochem.

[14] Qi P, Xu MD, Ni SJ, Huang D, Wei P, Tan C, Zhou XY, Du X (2013). Low expression of LOC285194 is associated with poor prognosis in colorectal cancer. J Transl Med.

[15] Tong YS, Zhou XL, Wang XW, Wu QQ, Yang TX, Lv J, Yang JS, Zhu B, Cao XF (2014). Association of decreased expression of long non-coding RNA LOC285194 with chemoradiotherapy resistance and poor prognosis in esophageal squamous cell carcinoma. J Transl Med.

[16] Cong M, Li J, Jing R, Li Z (2016). Long non-coding RNA tumor suppressor candidate 7 functions as a tumor suppressor and inhibits proliferation in osteosarcoma. Tumour Biol.

[17] Wang Y, Liu Z, Yao B, Dou C, Xu M, Xue Y, Ding L, Jia Y, Zhang H, Li Q, Tu K, Jiao Y, Liu Q (2016). Long non-coding RNA TUSC7 acts a molecular sponge for miR-10a and suppresses EMT in hepatocellular carcinoma. Tumour Biol.

[18] Shang C, Guo Y, Hong Y, Xue YX (2016). Long Non-coding RNA TUSC7, a Target of miR-23b, Plays Tumor-Suppressing Roles in Human Gliomas. Front Cell Neurosci.

[19] Ding YC, Yu W, Ma C, Wang Q, Huang CS, Huang T (2014). Expression of long non-coding RNA LOC285194 and its prognostic significance in human pancreatic ductal adenocarcinoma. Int J Clin Exp Pathol.

[20] Wang C, Yu J, Han Y, Li L, Li J, Li T, Qi P (2016). Long non-coding RNAs LOC285194, RP11-462C24.1 and Nbla12061 in serum provide a new approach for distinguishing patients with colorectal cancer from healthy controls. Oncotarget.

[21] Bian D, Shi W, Shao Y, Li P, Song G (2017). Long non-coding RNA GAS5 inhibits tumorigenesis via miR-137 in melanoma. Am J Transl Res.

[22] Shi L, Peng F, Tao Y, Fan X, Li N (2016). Roles of long noncoding RNAs in hepatocellular carcinoma. Virus Res.

[23] Bartonicek N, Maag JL, Dinger ME (2016). Long noncoding RNAs in cancer: mechanisms of action and technological advancements. Mol Cancer.

[24] Dong J, Xu J, Wang X, Jin B (2016). Influence of the interaction between long noncoding RNAs and hypoxia on tumorigenesis. Tumour Biol.

[25] Xiao J, Hu CP, He BX, Chen X, Lu XX, Xie MX, Li W, He SY, You SJ, Chen Q (2016). PTEN expression is a prognostic marker for patients with non-small cell lung cancer: a systematic review and meta-analysis of the literature. Oncotarget.

[26] Xiao J, Zou Y, Chen X, Gao Y, Xie M, Lu X, Li W, He B, He S, You S, Chen Q (2016). The Prognostic Value of Decreased LKB1 in Solid Tumors: A Meta-Analysis. PLoS One.

